# Airlift bioreactor–based strategies for prolonged semi-continuous cultivation of edible *Agaricomycetes*

**DOI:** 10.1007/s00253-024-13220-4

**Published:** 2024-06-18

**Authors:** Federico Cerrone, Conor Ó Lochlainn, Tony Callaghan, Peter McDonald, Kevin E O’Connor

**Affiliations:** 1 BiOrbic Bioeconomy Research Centre, O’Brien Centre for Science (Science East), University College Dublin, Belfield Campus, Dublin, Ireland; 2 School of Biomolecular and Biomedical Sciences, University College Dublin, Belfield Campus, Dublin, Ireland; 3 Commercial Mushroom Producers, Units7/8 Newgrove Industrial Estate, Monaghan, Ireland; 4 Bioplastech Ltd NovaUCD, University College Dublin, Belfield Innovation Park, Dublin, Ireland

**Keywords:** Airlift fermentation, Filamentous fungi, Volumetric productivity, Bioactives

## Abstract

**Abstract:**

Submerged cultivation of edible filamentous fungi (*Agaricomycetes*) in bioreactors enables maximum mass transfer of nutrients and has the potential to increase the volumetric productivity of fungal biomass compared to solid state cultivation. These aspects are paramount if one wants to increase the range of bioactives (e.g. glucans) in convenient time frames. In this study, *Trametes versicolor* (M9911) outperformed four other *Agaricomycetes* tested strains (during batch cultivations in an airlift bioreactor). This strain was therefore further tested in semi-continuous cultivation. Continuous and semi-continuous cultivations (driven by the dilution rate, *D*) are the preferred bioprocess strategies for biomass production. We examined the semi-continuous cultivation of *T. versicolor* at dilution rates between 0.02 and 0.1 h^−1^. A maximum volumetric productivity of 0.87 g/L/h was obtained with a *D* of 0.1 h^−1^ but with a lower total biomass production (cell dry weight, CDW 8.7 g/L) than the one obtained at lower dilution rates (12.3 g/L at *D* of 0.04 and vs 13.4 g/L, at a *D* of 0.02 h^−1^). However, growth at a *D* of 0.1 h^−1^ resulted in a very short fermentation (18 h) which terminated due to washout (the specific *D* exceeded the maximum growth rate of the fungal biomass). At a *D* of 0.04 h^−1^, a CDW of 12.3 g/L was achieved without compromising the total residence time (184 h) of the fermentation. While the *D* of 0.04 h^−1^ and 0.07 h^−1^ achieved comparable volumetric productivities (0.5 g/L/h), the total duration of the fermentation at *D* of 0.07 h^−1^ was only 85 h. The highest glucan content of cells (27.8 as percentage of CDW) was obtained at a *D* of 0.07 h^−1^, while the lowest glucan content was observed in *T. versicolor* cells grown at a *D* of 0.02 h^−1^.

**Key points:**

• *The highest reported volumetric productivity for fungal biomass was 0.87 g/L/h.*

• *Semi-continuous fermentation at D of 0.02 h−1 resulted in 13.4 g/L of fungal biomass.*

• *Semi-continuous fermentation at D of 0.07 h−1 resulted in fungal biomass with 28% of total glucans*.

**Supplementary Information:**

The online version contains supplementary material available at 10.1007/s00253-024-13220-4.

## Introduction

Filamentous fungi of the *Basidiomycota* division (*Agaricomycetes* class) are a morphologically complex group of macrofungi which includes the mushroom forming fungi. They form interlocking networks of primary mycelia of different lengths with a complexly regulated sexuality (Kües 2015; Coelho et al. 2017) that eventually produce heterokaryotic specialised structures of secondary mycelia arranged in fruiting bodies (mushrooms); these structures are the evolutionary structure able to disperse airborne haploid spores after karyogamy and meiosis. These macrofungi are known to produce structural polysaccharides that have immunomodulatory and therapeutic properties (Municio et al. 2013). There are also tissue-specific immunomodulatory differences regarding α vs β-glucans due to their linearity or branching (Morales et al. 2020). The immunological response resides in the polysaccharide driven stimulation of the macrophages to express dectin-1 receptors and the consequent release of a subset of cytokines for lymphocyte recruitment and the establishment of an adaptive immunity response in the organism (Municio et al. 2013; Dos Santos et al. 2019; Moerings et al. 2021). Macrophage priming for adaptive immunity is also regulated by epigenetic changes and tissue-specific homeostatic controls (Glass and Natoli 2016). Gut microbiota stimulating polysaccharides and the interplay with the gut-associated lymphoid tissue (GALT) (Morbe et al. 2021) are specifically linked to the lowering of glucose levels in the blood stream, reduction of insulin resistance and lowering of (low-density lipoprotein) LDL cholesterol in mammals and in humans in particular. These polysaccharides are mainly constituted by repetitions of monomeric glucose units, linked by *O*-⍺ and β glycosidic bond in a 1,3 position and branched by 1,6 glycosidic bonds connecting adjacent chains of polymers. The degree of branching, the total molecular weight of the polymer and the conjugation with polypeptides are shown to have a key effect in enhancing the therapeutic potential of the compounds (Adams et al. 2008; Morales et al. 2020). Some of the most widely used edible fungal species known to produce glucan molecules are as follows: *Lentinula edodes* (Pan et al 2021), *Agaricus* (*blazei* and *bisporus*) (Huang et al. 2022) and *Hericium* sp. (Feng et al. 2019). Another glucan producing filamentous fungus, still edible but less palatable, is *Trametes versicolor* (Li et al 2019)*. T. versicolor* is a wood-rotting fungus that grows in temperate forests and in particular on deciduous logs or stumps of tree trunks (del Cerro et al. 2021). It has a characteristic fan (or turkey tail)-shaped morphology with a zoned variable (versi) multicoloured concentric appearance. Usually, the pigmentation of the fungal biomass is due to the production of a variety of compounds such as terphenylquinones, polyenes, phenazines and polyketides (Tauber et al. 2018). In addition to the production of bioactive compounds (Habtemariam 2020), *T. versicolor* has also been used for the bioremediation of phenol rich substrates (Cerrone et al. 2011) and therapeutic extracts have been used in clinical settings since the 1970s in Japan to treat cancer (Sullivan et al. 2006).

Wild-type growth of fungi (specifically the ones that belong to the Class of *Agaricomycetes* of the *Basidiomycota* division) (Wijayawardene et al. 2020) is associated with the formation of a basidiocarp or mushroom (fruiting body) for the production and release of haploid spores (Kües and Navarro-González 2015; Nagy et al. 2023). *Lentinula* and *Agaricus* show a more characteristic cap-mushroom type of fruiting body with a brown (*Lentinula*), beige (*A. blazei*) or white (*A. bisporus*) hue respectively. The *Hericium* genus displays a shiny white fruiting body that has been described as an icicle or wisteria-like type of flowering plant (Boddy et al. 2011).

Submerged cultivation of mycelial clumps in a suitable growth medium in flasks or bioreactor vessels, excludes the formation of the fruiting body. Fungi grown in submerged cultivation aggregate in mycelia clumps of different thickness, depending on the phase of growth (Veiter et al. 2018). The submerged cultivation strategy is a potentially efficient method to grow filamentous mycelia with higher volumetric productivity and increase biomass yields (g of cell dry weight (CDW)/g of C source). With this strategy, solid substrate–based month-long cultivations can be reduced to days since the mycelia can quickly transport nutrients through their cell walls and do not have to extend the filamentous hyphae to uptake nutrients from the surrounding environments (Intasit et al. 2021). On the other hand, the pelletised aggregation of mycelia reduces oxygen mass transfer and oxygen solubility and so increases the chances of oxygen deprivation inside the core of the pellets (Patel et al. 2023). The hyphal aggregates are especially prone to shear stress damage because of the entanglement of the filamentous morphology when they are propagated in a bioreactor that has a high stirring speed (Shahryari and Niknezhad 2023). A continuous cultivation fermentation strategy could allow high throughputs if the fungus is able to sustain high dilution rates, but it has the drawback of a lower cell dry mass and it can only be sustained for a minimal retention time which can lead to a reduced total duration of the bioprocess (Patel et al. 2023). Both the extension of the duration of the continuous bioprocess and the mass of the accumulated biomass are key for a bioprocess that could achieve sufficient productivity and scalability. The control of the mycelial morphology through bioreactor design and contemporary fermentation strategy is therefore both of paramount importance (Osadolor et al. 2018). Minimising biomass adhesion to bioreactor components (e.g. pH and dissolved oxygen (DO) probes, stirrer and walls (Nieland et al. 2021)) is a key challenge to address when growing filamentous fungi in a bioreactor. In this study, we utilise a dilution rate-based approach to maximise the fungal biomass production inside an airlift bioreactor (that maintained a minimal level of shear stress) adopting a semi-continuous fermentation strategy in an attempt to maximise biomass productivity and b-glucan content and to prolong the duration of the fermentation.

## Materials and methods

### Culture conditions, media and flask experiments

Four individual fungal (*Basidiomycetes*) strains were purchased from Mycelia®, Deinze, Belgium; they were obtained as slanted media of malt agar with an actively growing mycelia covering the surface. Those strains were as follows: *T. versicolor* (M9911), *L. edodes* (M3102),* H. erinaceus* (M9514) and *A. blazei* (M7700)*. A. bisporus* (substrain White, ABS14) was obtained from Commercial Mushroom Producers, Monaghan, Ireland. The strains were grown on malt agar plates according to the specifications provided by Mycelia® Deinze, Belgium, and were incubated at 28 °C. The starting flasks cultures were prepared as follows: five individual agar plugs covered with an actively growing mycelia were taken from each plate aseptically. The same were transferred into a 100-mL flask, provided with a stainless-steel spring to facilitate mycelia attachment. The medium composition of the flask was the following: 3 g/L of yeast extract, 5 g/L of soy peptone, 3 g/L malt extract and 10 g/L of glucose; all the reagents were of analytical grade, and they were purchased from Sigma-Aldrich, Dublin, Ireland. Each flask final volume was 100 mL, and they were autoclaved at 121 °C for 15 min before being inoculated. The medium was called YSMG. After a minimal period of 5 days of growth and with a dense attached mycelial aggregate on the stainless-steel spring, the starter culture was used to inoculate the experimental cultures, where the same or a different range of media was used. In order to facilitate a dense and homogenous pelletised fungal population, an IKA® T18 UltraTurrax® submergible homogeniser (Staufen, Germany) provided with a S18 N 10G dispersing tool, able to reach a maximum speed of 25,000 revolutions per minute (RPM), was used. The mycelial aggregate, present in the starting flask, was homogenised for 30 s in aseptic conditions inside a SafeFast laminar cabinet (produced by Faster S.r.l, Ferrara, Italy) by the submergible homogeniser. The flask was inclined at an angle of approximately 45° for the duration of the homogenisation. The experimental flasks cultures and the inoculation cultures for the airlift bioreactor were also prepared in this way. All experimental flasks and airlift bioreactors were kept at 25 °C. Flasks were incubated with a shaking regime of 150 RPM.

### Long-term storage of fungal cultures

The different *Basidiomycetes* strains were grown in cultivation flasks in the above-mentioned media for 5 days, until a homogenous population of pellets developed an approx. size of 3 mm. Pelletised aggregates of the different *Basidiomycetes* strains cultivated in flasks were homogenised in sterile conditions using an IKA® T18 UltraTurrax® submergible homogeniser (Staufen, Germany) provided with a S18 N 10G dispersing tool. After homogenization, 100 μL of the culture was transferred into sterile cryovials already containing 450 μL of sterile glycerol (60% v/v solution) and 450 μL of sterile sucrose (30% w/v solution) for a final volume of 1 mL. The cryovials were kept in an ice bath at 0 °C for 5 min before being transferred into a − 70 °C Thermo Scientific Revco® Elite Plus® freezer (Dublin, Ireland).

### Airlift bioreactor experiments

**Fig. 1:**
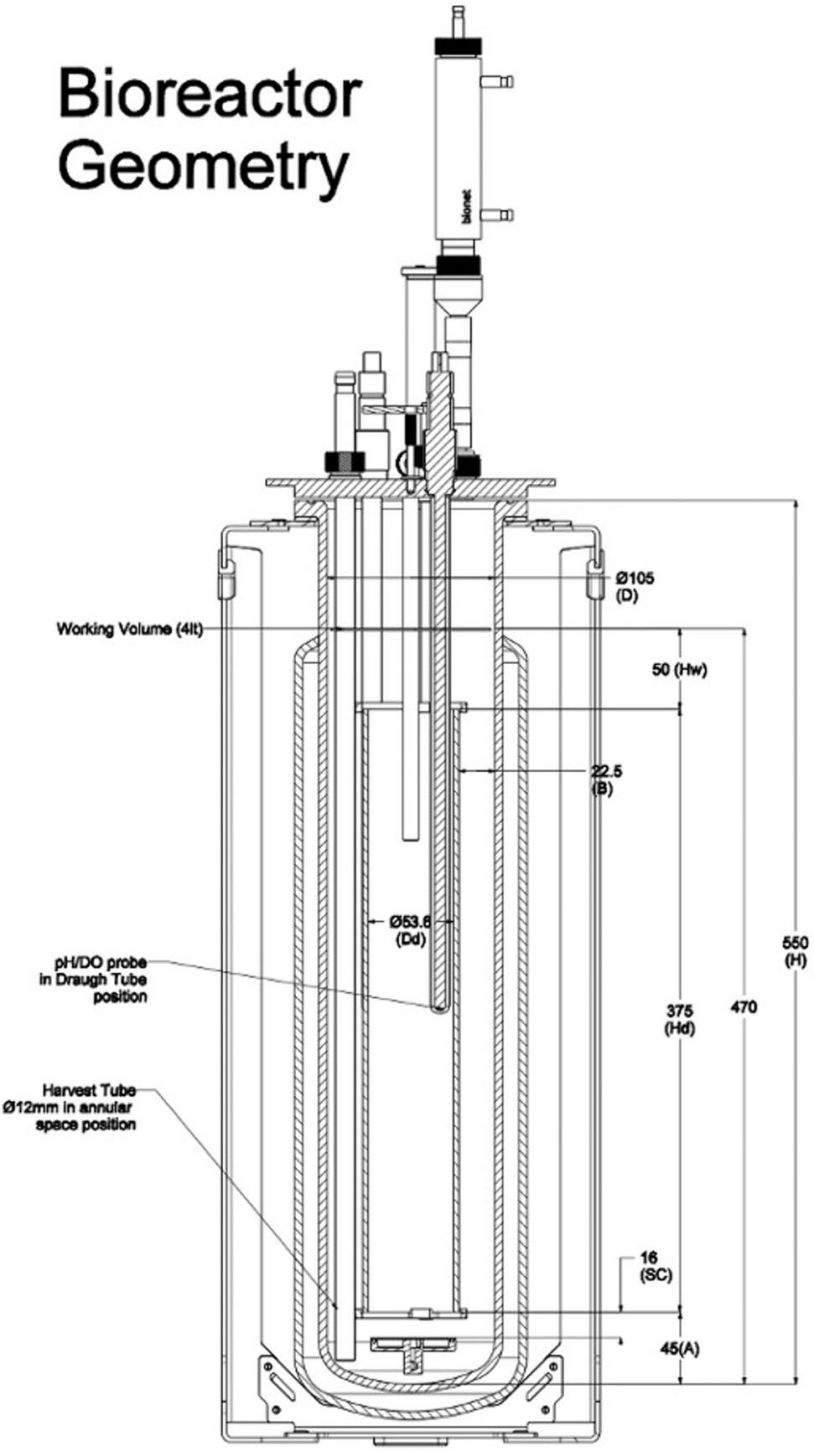
Diagram picture of the Bionet® airlift bioreactor (Fuente Alamo de Murcia, Spain) equipped with an internal draught tube and a bottom stainless-steel ring sparger

The airlift bioreactor was custom-manufactured regarding the design, software control and continuous module by Bionet®, Fuente Alamo de Murcia, Spain. A twin platform system of two identical airlift bioreactors was used. The bioreactor is shown in the picture diagram (Fig. 1).

The bioreactor was a double jacketed transparent and cylindrical vessel; it had a working volume of max 5 L, and it had an internal draught tube (length of 260 mm with a diameter of ∅ 40 mm) over a stainless-steel ring sparger provided with ∅ 1 mm holes. The advective-like airlift movement was guaranteed by the bioreactor elongated design, and the resulting gradient in hydrodynamic density allows the pelletised biomass and the media nutrients to completely recirculate in 17 s approximately descending inside the annular exterior ring (downcomer) and raising inside the draught tube. The air sparging provided with a filtered mechanism was maintained at a minimal 1 vvm. The continuous process module, a custom-made unit by Bionet® (Fuente Alamo de Murcia, Spain), is equipped with two peristaltic pumps of different heads (one smaller for the addition of the fresh medium and one bigger for the withdraw of the spent medium and the grown biomass). The two pumps worked in a cyclical fashion to match the hourly controlled dilution rate, typically working at a very fast pace for the medium withdrawing pump and at a very fine-tuned and slow flow rate for the medium adding pump. The medium for both the batch and the semi-continuous fermentations was the following: glucose, 10 g/L, yeast extract, 3 g/L (purchased from Sigma-Aldrich, Dublin, Ireland) and corn steep liquor (CSL)15 mL/L (purchased from Santa Cruz Biotechnology, Heidelberg, Germany). Each batch and semi-continuous airlift fermentation was performed in triplicate.

### Analytical procedures

Glucose and other monosaccharide analyses were detected by using a Shimadzu® HPLC equipment (Duisburg, Germany) provided with a RID detector. The mobile phase used was 0.014 N H_2_SO_4_ in a Milli-Q aqueous solution that was previously vacuum filtered with 0.2-μm filter pore size; this mobile phase was used in an isocratic fashion. The column used was a BioRad® Aminex HPX-87H (Watford, England) with a solid polymeric matrix composed of polystyrene divinylbenzene. The flow rate of the mobile phase was 0.55 mL/min. α and β-glucans were detected by an enzymatic-based colorimetric method using a specific Megazyme® yeast/fungi glucan detection kit (Wicklow, Ireland) following the company provided protocol.

## Results

### Media optimisation for the growth of mycelia pellets in flasks

**Fig. 2:**
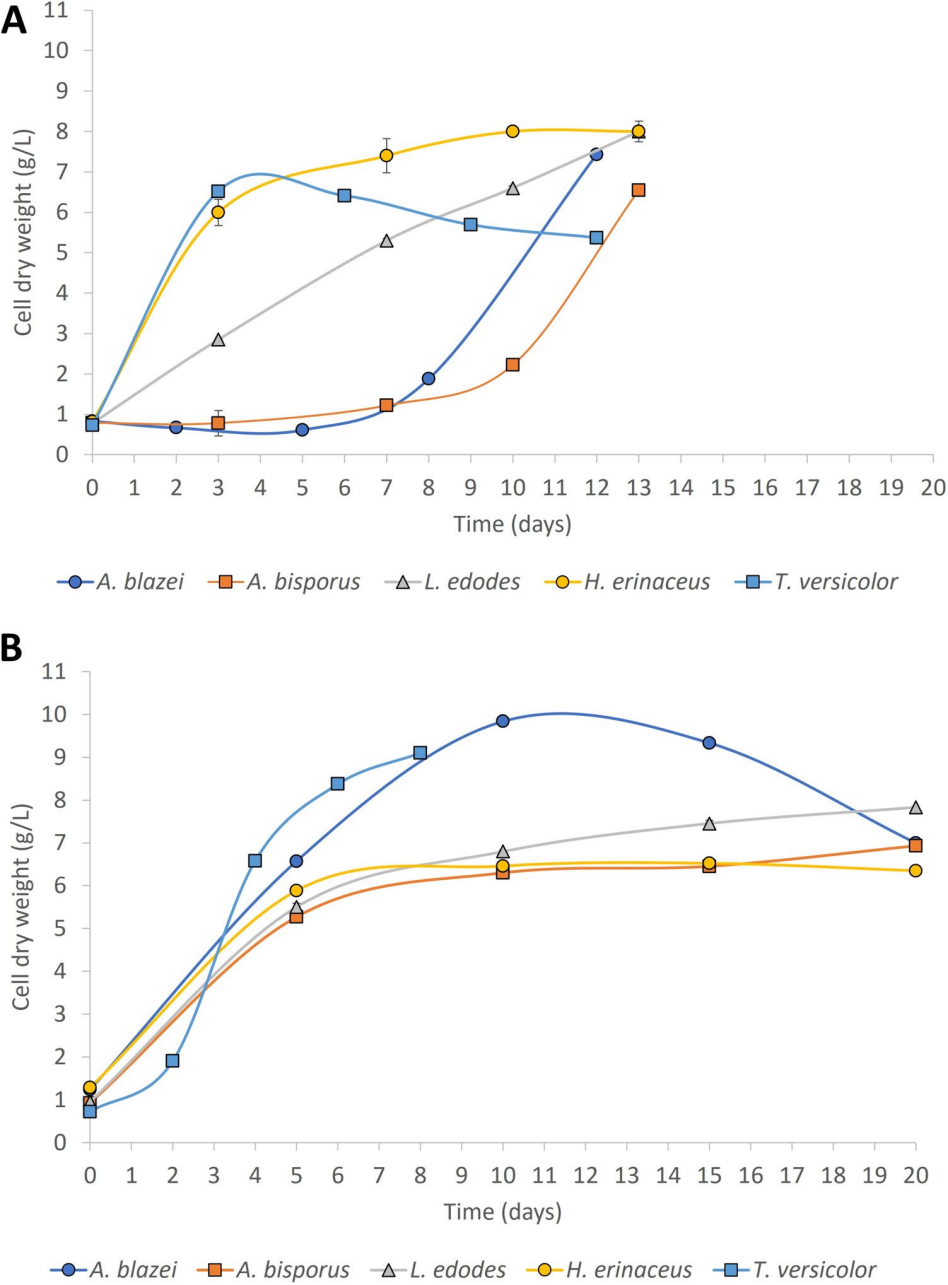
**A** Selected *Agaricomycetes* strains growth curves in YSMG media in shaken flask experiments at 25 °C and 150 RPM. **B** Selected *Agaricomycetes* strains growth curves in CSL media in shaken flask experiments at 25 °C and 150 RPM. Each set of cultures was grown in triplicate experiments

Homogenised seed cultures of *A. blazei*, *A. bisporus*, *T. versicolor*, *L. edodes* and *H. erinaceus* grown in YSMG medium were transferred to the same fresh medium (5% v/v inoculum). The strains grew in a pelletised form, nucleating around shredded mycelia after varying number of days, depending on the fungal strain. Each strain achieved a unique maximum cell dry weight (CDW) (Fig. 2A); this was achieved over a range of incubation times starting from a comparable sized inoculum. *T. versicolor* showed the highest biomass production at the earliest time point (6.5 g/L, after 3 days); after that, the CDW declined by a maximum 18%. *H. erinaceus* showed the highest biomass value (8 g/L) but only after 10 days of growth. *L. edodes* showed a linearly increasing trajectory but reached the same CDW as *H. erinaceus* after 13 days of growth. *A. blazei* and *A. bisporus* both had a long lag phase and only started to increase their CDW after 8 and 10 days respectively; their maximum CDWs were 7.4 and 6.6 g/L. Subsequently, a corn-steep-liquor amended medium was designed, similar to Huang et al. (2007). The fungal strains grew differently in CSL media compared to YSMG. *T. versicolor* showed a diminished lag phase and achieved 6.6 g/L of biomass after 4 days of growth. *A. blazei* showed the highest biomass but only after 10 days of growth. All the other three strains (*L. edodes*, *H. erinaceus*, *A. bisporus*) grew very similarly in CSL media, with *L. edodes* having 7.8 g/L of biomass after 20 days of growth (Fig. 2B). After this preliminary set of experiments, a systematic approach of removing single components from this CSL medium was undertaken to try to design the cheapest and most minimal medium possible for bioreactor experiments.

### Media component reduction experiments

**Fig. 3:**
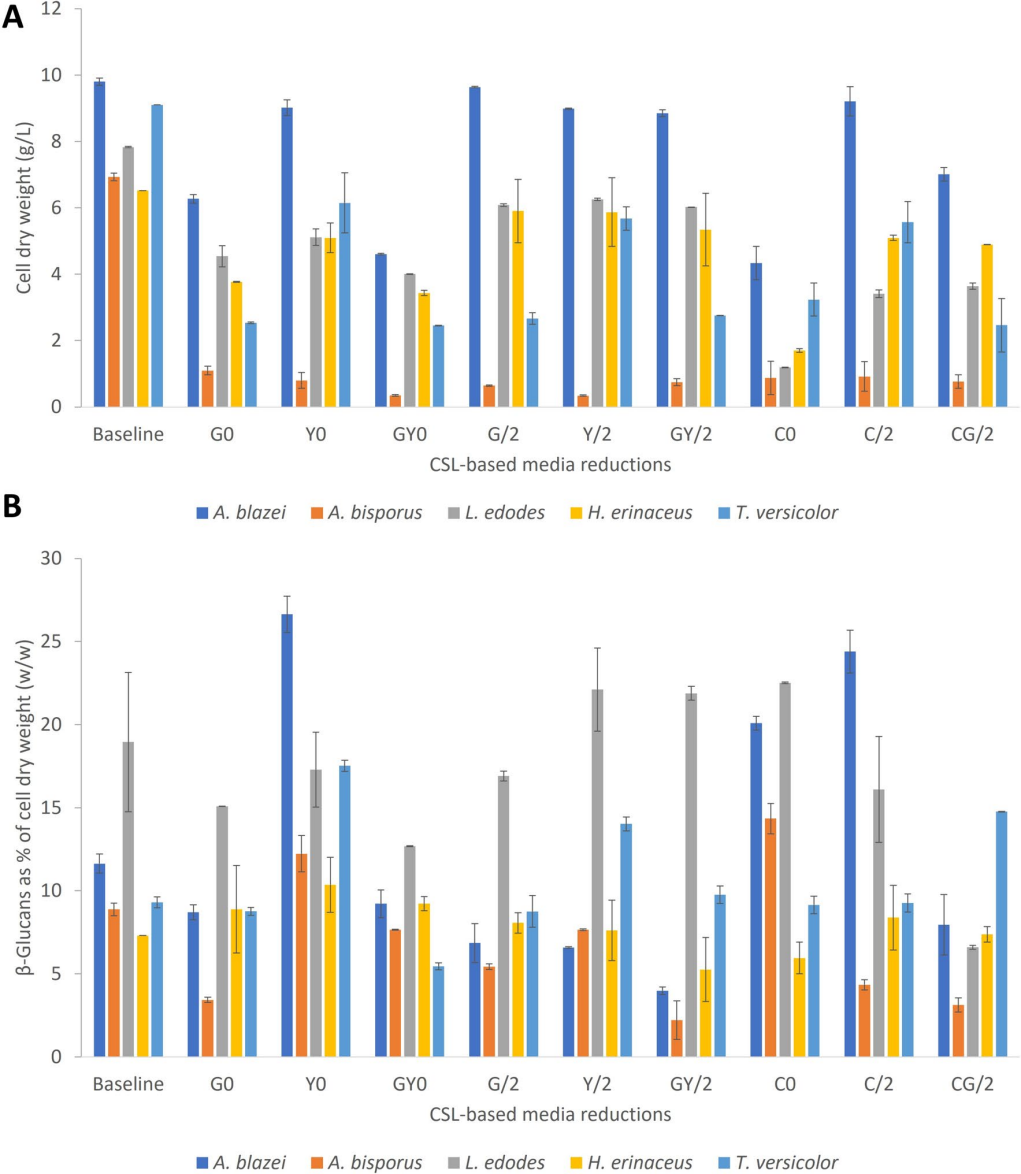
**A** Fungal biomass in CSL media reductions. **B** β-glucans (as percentage of CDW) produced by fungal biomass in CSL media reductions. Baseline: glucose, G 10 g/L; yeast extract, YE 3 g/L; corn steep liquor, CSL 15 mL/L. G0 = same as baseline but no glucose; Y0 = same as baseline but no yeast extract; GY0 = same as baseline but no glucose and yeast extract; G/2 = same as baseline but glucose = 5 g/L); Y/2 = same as baseline but YE 1.5 g/L; GY/2 = same as baseline but halved glucose and yeast extract (5 and 1.5 g/L respectively); C0 = same as baseline but no CSL; C/2 = same as baseline but halved CSL (7.5 mL/L); CG/2 = same as baseline but halved CSL and glucose (7.5 mL and 5 g/L respectively). Each media condition was tested in triplicate experiments

The comprehensive set of experiments targeting fungal biomass production with media component reduction is shown in Fig. 3 (Fig. 3A displays the biomass production, while Fig. 3B exhibits the percentage of the CDW made up of the structural β-glucans that each respective strain produced under the same conditions).

The removal of glucose (G0, Fig. 3A) as a carbon source caused a reduction of the CDW values of *L. edodes*, *H. erinaceus* and *T. versicolor* by 42, 42 and 72% respectively; the removal of yeast extract decreased biomass by between 8 and 34% (Y0, Fig. 3A). Very similar CDW reductions were seen when both yeast extract and glucose were completely removed from the medium (Fig. 3A). Reducing glucose or yeast extract concentrations (singularly or together) by 50% (G/2, Y/2, GY/2) showed a severe impact on *A. bisporus* and *T. versicolor* (Fig. 3A); *T. versicolor* biomass decreases by 71 and 70%, when glucose or glucose/yeast extract were halved respectively. Significant but lower reductions of fungal dry weight (< 23%) for *L. edodes* or *H. erinaceus* were observed for these “50%” media component reductions.

The removal of CSL (C0, Fig. 3A) from the growth medium has the most severe impact on the growth of *L. edodes* with biomass decreased by 84%, but *H. erinaceus* and *T. versicolor* were also significantly impacted with a reduction of cell dry weight by 73 and 64% respectively. Restoring half of the CSL concentration (C/2, Fig. 3A) allows each strain to recover their biomass, but it showed still a marked impact on *L. edodes* biomass (56% lower than baseline levels). *H. erinaceus* is the most resilient strain in surviving the lack of primary carbon source (halving of glucose concentration) and secondary carbon source (CSL). This strain shows a 25% reduction in CDW when grown under those conditions. The reduction of critical components has a varied effects on the total glucan production by the selected fungal strains.

#### Airlift bioreactor fungal growth operated in batch mode

**Fig. 4:**
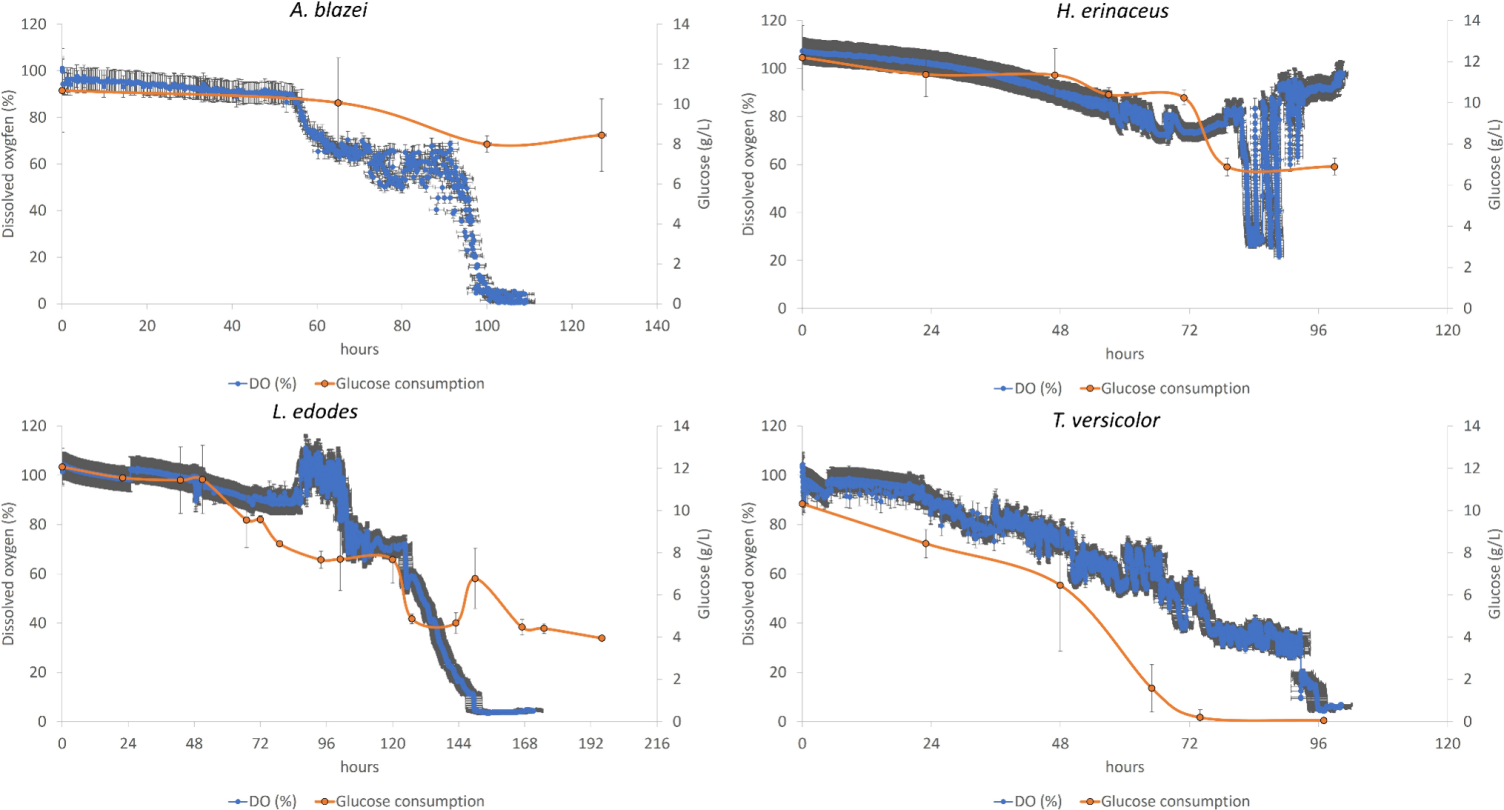
Glucose consumption and dissolved oxygen trend by the four selected fungal strains (*A. blazei*, *H erinaceus*, *L. edodes* and *T. versicolor*) in the airlift bioreactor in batch mode. Each plotted batch is an average of triplicate experiments

Four different *Basidiomycota* strains were grown in airlift bioreactors with CSL (baseline composition)-amended media. *A. bisporus* was not brought forward due its inability to grow in reduced nutrient medium. The airlift bioreactor has an internal draught tube that allows the uplift of the sparged air, and the downcomer conveys the descending compressed air because of a gradient of density; this allows a continuous recirculation of the resident suspended biomass, and this complete loop movement takes approx. 17 s if not impeded, but the different densities of the pelletised biomass (strain-specific and/or because of the stage of growth) play a considerable and dynamic role. The fungal strains grown in the airlift were *A. blazei*, *H. erinaceus*, *L. edodes* and *T. versicolor*. All of them showed a different substrate consumption rate when grown in batch mode (Fig. 4). Taking 100 h as a reference point for the late exponential phase of growth of the strains, we can clearly differentiate the behaviour of the specific fungal strains, regarding glucose consumption. *A. blazei*, *L. edodes* and *H. erinaceus* started consuming detectable amount of glucose only between 65 and 100 h of growth, but while *H. erinaceus* had consumed 44% of the available glucose at 100 h (with a consumption of the remaining 15% in the last 23 h of growth (T100 to T123)), *A. blazei* and *L. edodes* consumed 25% and 36% of their available glucose, respectively, at the same time point (100 h). After that point, *L. edodes* increased its glucose consumption, consuming 64% of the total available glucose (40% of it in the last 96 h of growth); *A. blazei* stopped consuming glucose after 100 h possibly because of the lack of critical nutrient such as amino acids that impaired the growth of this specific strain. *T. versicolor* consumed 100% of the available glucose in the first 100 h of growth, showing the consumption of 14% of glucose in the first 48 h of growth and the remaining 86% amount of glucose in the following 52 h. The substrate consumption rate by *T. versicolor* is 2- to 4.43-fold higher than *H. erinaceus*, *L. edodes* and *A. blazei* respectively. The highest substrate consumption rate by *T. versicolor* is between 48 and 97 h of growth (0.133 g/L/h) and that is twofold higher than the second highest rate (by *H. erinaceus*, between 99 and 122 h) or 24-fold higher than the lowest substrate consumption rate (by *A. blazei*, between 0 and 65 h). The *μ*_MAX_ of *T. versicolor* in batch cultivation in the airlift bioreactor was 3-, 9- and 11.8-fold higher than the *μ*_MAX_ of *H. erinaceus*, *L. edodes* and *A. blazei* respectively.

**Fig. 5:**
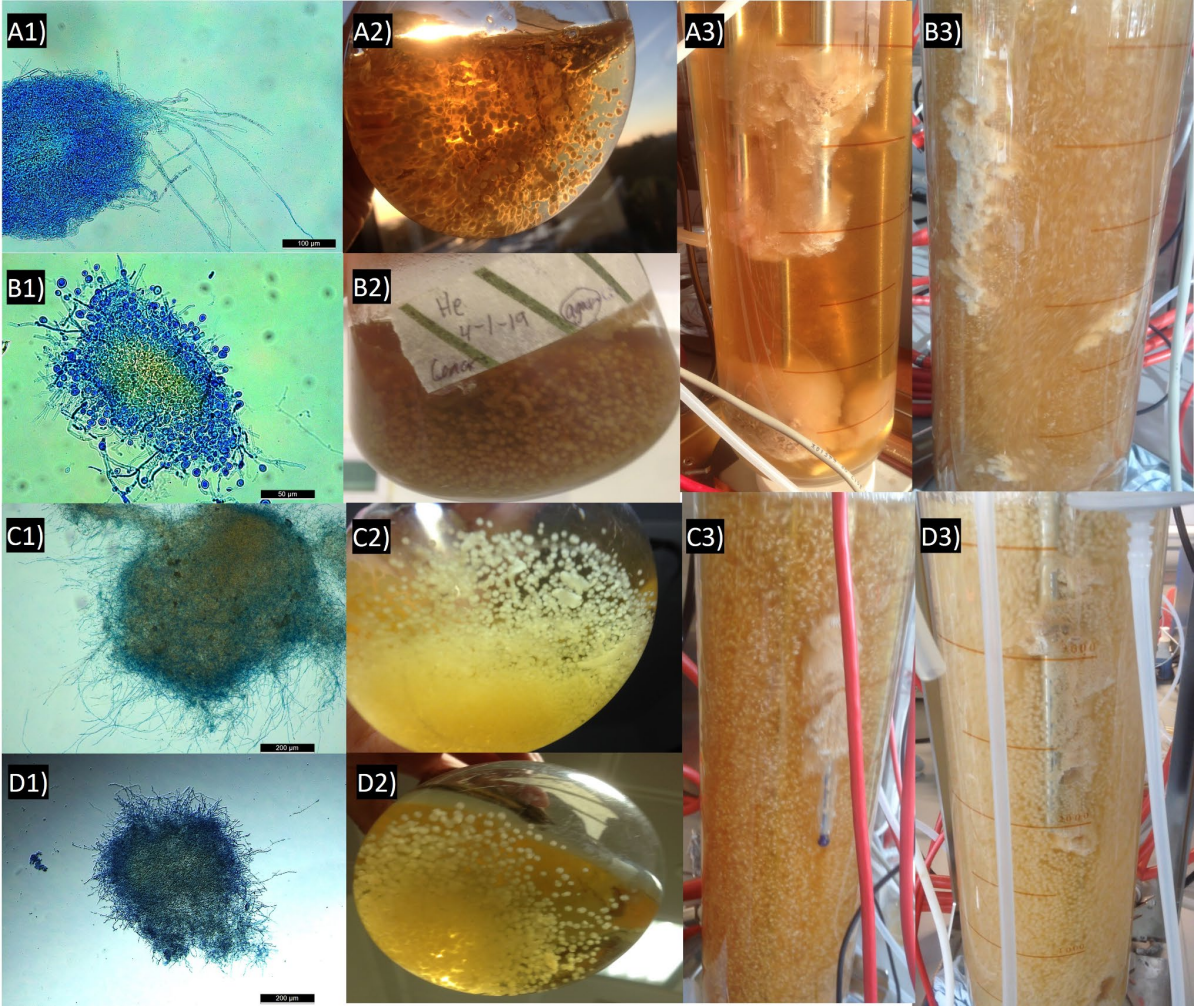
(A1–3) Microscopic mycelial pellet of *A. blazei*, pelletised aggregation of *A. blazei* in the seeding flask, pelletised aggregation of *A. blazei* in the airlift bioreactor. (B1–3) Microscopic mycelial pellet of *H. erinaceus*, pelletised aggregation of *H. erinaceus* in the seeding flask, pelletised aggregation of *H. erinaceus* in the airlift bioreactor. (C1–3) Microscopic mycelial pellet of *L. edodes*, pelletised aggregation of *L. edodes* in the seeding flask, pelletised aggregation of *L. edodes* in the airlift bioreactor. (D1–3) Microscopic mycelial pellet of *T. versicolor*, pelletised aggregation of *T. versicolor* in the seeding flask, pelletised aggregation of *T. versicolor* in the airlift bioreactor. The bar size in Figure A1 is 100 μm; in Figure B1, it is 50 μm; and in Figures C1 and D1, it is 200 μm respectively.

Dissolved oxygen trends of each individual fungal strain mirror the consumption of substrate (Fig. 4). This is not surprising but what is surprising is the fact that *A. blazei* showed a high oxygen consumption without the concomitant glucose consumption, while the opposite occurs for *H. erinaceus*. In fact, *H. erinaceus* shows an initial minimal oxygen consumption (22% drop in the dissolved oxygen in the first 84 h, corresponding to consumption of 33% of the available glucose) and the consumption of glucose after this point results in highly elevated oxygen consumption. After that, the strain stopped the carbon consumption, also evident from the rise of the DO (%), indicating an arrest of metabolic activity. *L. edodes* and *T. versicolor* have a more gradual oxygen consumption trend that exactly reflects their slower and faster substrate consumption rate respectively (Fig. 4). Microscopic and macroscopic aggregates of mycelia pellets of the four different fungal strains in the seeding flasks and in in the airlift bioreactors (batch mode) are shown in Fig. 5. Regarding the strain specific glucan production, this was analysed at the end of the batch phase of each of the four fungal strains (Table 1). Seen from the performance of *T. versicolor* in the batch cultivation in the airlift bioreactor, especially for the highest *μ*_MAX_, higher substrate consumption rate and the highest β-glucans/total glucans ratio, this strain was brought forward for semi-continuous fermentations studies.

**Table 1:** Glucan production by the selected fungal strains in the airlift bioreactor at the end of their respective batch phase

Strain	*A. blazei*	*L. edodes*	*H. erinaceus*	*T. versicolor*
Compound	% of CDW*	% of CDW	% of CDW	% of CDW
Total glucans	13.4 ± 4.7	18.4 ± 2.8	19.6 ± 2.3	10.6 ± 0.4
α-glucans	8.3 ± 3.7	4.4 ± 3.4	6.2 ± 2.7	0.7 ± 0.5
β-glucans	5.1 ± 2.2	14.0 ± 2.0	13.4 ± 4.6	9.9 ± 0.9

*Glucan content as a percentage of cell dry weight (CDW)

#### Airlift-based fungal growth operated in semi-continuous fermentations

Although *T. versicolor* does not have the highest glucan content, it has the highest growth rate of all four strains with an effective substrate consumption rate (Table 1; Fig. 4). For these reasons, this strain underwent a series of semi-continuous fermentations to determine how far we could push the strain to achieve high biomass, high biomass yield (g CDW/g substrate) and maximise the dilution rate while sustaining biomass generation (i.e. avoiding wash out). The semi-continuous set up of the fermentation reflects the nature of the fungal biomass which grow as pellets; to allow for withdrawal of this biomass, a wide bore tube (16 mm) is needed. Liquid medium delivery requires a narrow (1.6 mm) bore tube. Both medium addition and withdrawal are achieved using peristaltic pumps. The two pumps work in combination; a slower pump delivers the medium over a prolonged period of time (minutes), while the faster pump works to withdraw the medium over a very short period of time (seconds).

In submerged conditions, the fungal biomass grows according to an exponential equation until the density of the pellet causes limitation of oxygen/nutrients transfer to the fungal biomass; this has only been verified at the actively dividing tip of the hyphal ends of the spherical pellets. In its entirety, the pellets grow according to a linearly increasing cubic root growth model (Pirt 1966; Cui et al 2000), following the equation:

$${M}_{t}^{1/3}=kt+{M}_{0}^{1/3}$$

where *M*_0_ is value of concentration of biomass at time 0 and *M*_*t*_ is the concentration of the biomass at time, *t*. *k* is a constant (g^1/3^/L^1/3^/day), and *t* is the specific unit of time.

This means that the cubic root values of the increasing values of biomass would be only interpolated by a linearly increasing curve. The growth rate curve for this specific strain also identifies that the maximum doubling time of this strain is between 12 and 17 h. A stepwise approach to gradually increase the dilution rates in multiple rounds of fermentations was undertaken. The first tested dilution rate was 0.02 h^−1^_._

### *D* 0.02 h.^−1^ (with a 48-h batch phase)

**Fig. 6:**
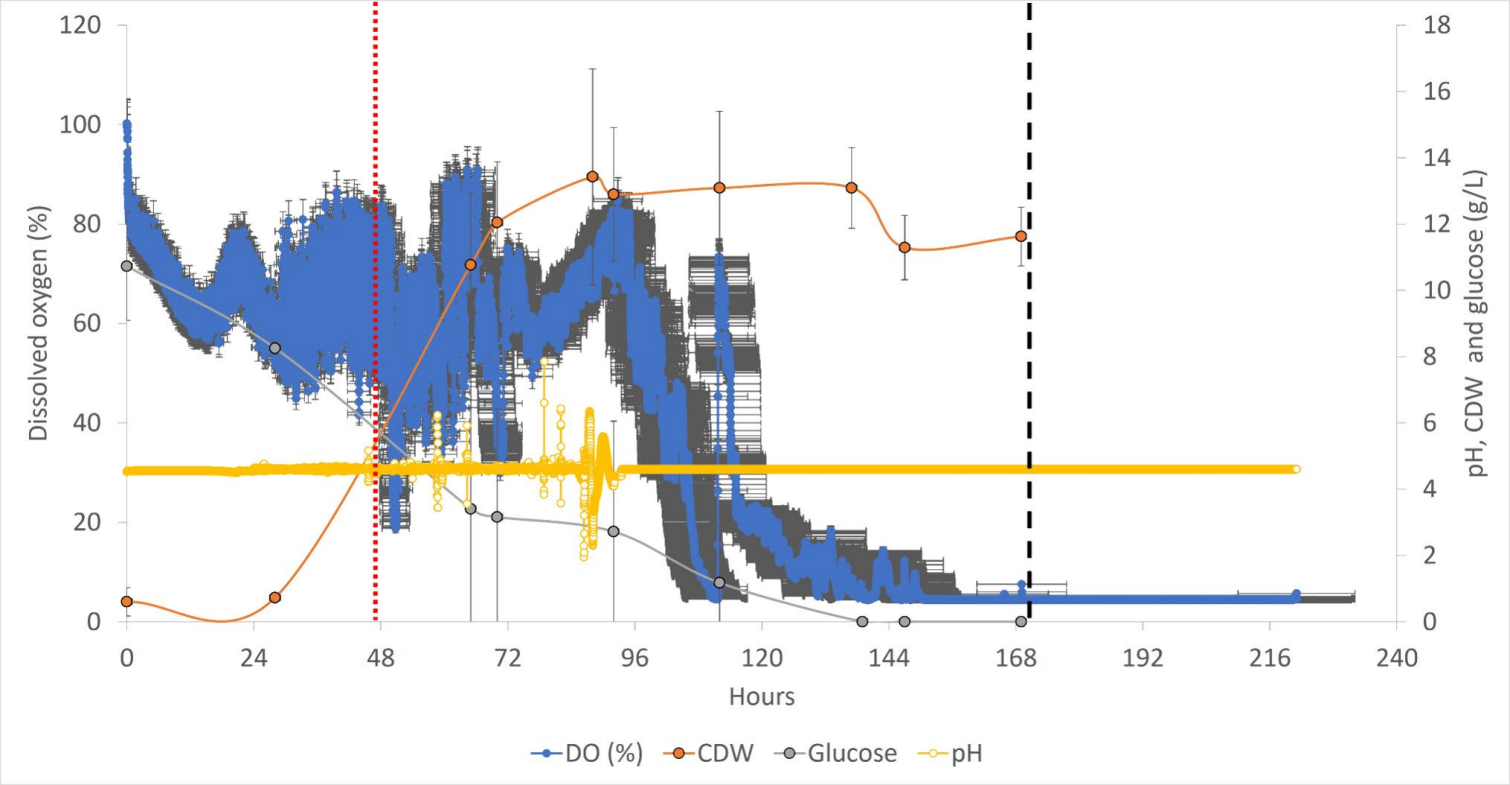
Average plot of triplicate semi-continuous fermentations targeting a dilution rate (*D*) of 0.02 h^−1^ for the growth of *T. versicolor* after a batch phase of 48 h. A red vertical dotted line indicates the end of the batch phase and therefore the start of the semi-continuous feeding regime in the semi-continuous fermentations. A vertical black dashed line identifies the onset of macroaggregate pellet formation

A pellet-rich dense 5-day-old culture of *T. versicolor*, grown in a shake flask of 800 mL, was added as the inoculum to a 4-L airlift bioreactor after aseptic homogenisation with an IKA® T25 Digital Ultraturrax® homogeniser provided with a S25KD 18G ST dispersing tool operated at 25000 RPM for 30 s. The inoculum was added to the bioreactor using the peristaltic pump that would be used later for the addition of the fresh medium at the end of the batch phase; the end of the batch phase was hypothesised to occur due to a consistent dissolved oxygen increase that interrupted a downward trend of oxygen consumption at 48 h. One hundred sixty millilitres of fresh medium was added to the bioreactor every 2 h (80 mL/h). The first addition of fresh medium started at 48 h, and that was followed by a dissolved oxygen drop until 60 h where a strong sustained rise of the dissolved oxygen occurred. The dissolved oxygen started rising after that and stayed at an average value of 80% for approximately 6 h (with pronounced oscillations), likely due to an unexplained sudden rise in the pH value that was promptly corrected by the buffering system; after that period of time, the usual feeding regime allowed the dissolved oxygen to be consumed as normal, likely because the foaming, caused by the pH rise, subsided. The oxygen consumption showed that this dilution rate (*D*) was optimal for the fungal growth, especially after 88 h, when the maximum CDW was obtained; this CDW was maintained for at least a further 80 h, when the semi-continuous fermentation was stopped. The main reason for stopping was because macroaggregates of mycelia were seen adhering to the glassware surfaces. The average maximum CDW obtained with these fermentation strategies was 13.42 g/L, and the yield was 0.38 g of biomass/g of glucose with a volumetric productivity of 0.26 g/L/h. A maximum CDW of 15.7 g/L was also obtained with a semi-continuous fermentation with a *D* of 0.02 h^−1^ after a batch phase of 65 h (data not shown), but a decrease of the volumetric productivity was also seen because of this longer duration; the extension of the batch phase up to more than 65 h was adopted for higher dilution rates (*D* of 0.07 and 0.1 h ^−1^) where the risk of washing out is increased, and therefore, a denser hyphal population has to be produced in the batch phase; this dense fungal population can then cope with higher feeding regimes. Establishing the range of duration of the intended batch phases, the following logical step was to increase the dilution rate of the semi-continuous fermentation (Fig. 6).

### *D* 0.04 h^−1^ (with a 31-h batch phase)

Building on the initial success of growth at dilution rate of 0.02 h^−1^, a range of fermentations was attempted at a dilution rate of 0.04 h^−1^. The first semi-continuous fermentation started with a batch phase of 31 h, followed by an addition of fresh medium at a flow rate of 160 mL/h and a harvest of pelletised biomass and spent medium at the same flow rate. A slight glucose accumulation was witnessed in the first 20 h of the semi-continuous phase (31 to 51 h); the glucose concentration rose from a value of 8.23 g/L to a value of 8.88 g/L. This equates to a glucose accumulation rate of 0.025 g/L/h (Supplemental Fig. S1). After this initial phase, the biomass is able to cope with the dilution rate and starts actively to consume the added glucose (Supplemental Fig. S1); this is reflected both by the decrease of the dissolved oxygen and by the drop of the glucose concentration inside the vessel. The percentage of dissolved oxygen drops by 44%, and the glucose concentration drops by 46% (a constant consumption rate of glucose of 0.25 g/L/h). This metabolically active phase lasts for the following 56 h (from 44 to 100 h). After this period, the fungal biomass starts to accumulate as the usual macro-aggregates and enters into a resting phase that is reflected by an accumulation of glucose at a rate of 0.049 g/L/h (from 110 to 140 h). In the last 30 h of the semi-continuous fermentation (110 to 140 h), glucose is consumed at a rate of 0.032 g/L/h and a concomitant accumulation of fungal biomass in the vessel that rises to a value of 7.31 g/L (Supplemental Fig. S1). The overall volumetric productivity is similar to what was achieved with a *D* of 0.02 h^−1^ (0.29 g/L/h vs 0.31 g/L/h), but the biomass yield was only 0.17 g/g (approximately 30% lower than that achieved at a *D* of 0.02 h^−1^).

### *D* 0.04 h^−1^ (with a 48-h batch phase)

**Fig. 7:**
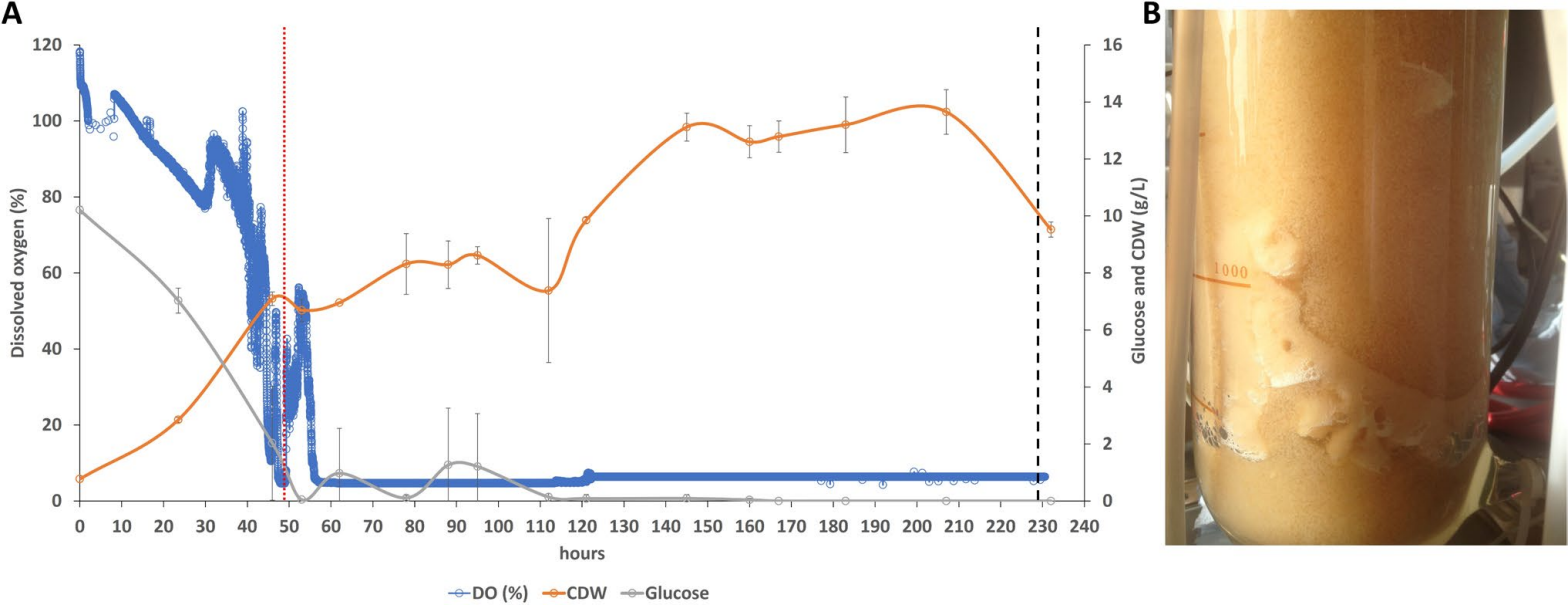
**A** Average plot of triplicate semi-continuous fermentations targeting a dilution rate (*D*) of 0.04 h^−1^ for the growth of *T. versicolor* after a batch phase of 48 h. A vertical red dotted line identifies the start of the feeding regime, while the vertical black dashed line identifies the onset of macroaggregates pellets formation. **B** Evidence of mycelia aggregates in airlift bioreactor after a semi-continuous fermentation with a *D* of 0.04 h^−1^

In light of the limited glucose consumption witnessed in the batch phase (1.7 g/L), the batch phase was extended from 31 to 48 h. The three repetitions showed better consumption of glucose in 48 h (batch phase compared to the experiment that had a 31-h batch phase). The glucose concentration went to zero at the end of the 48-h batch fermentation and stayed at that value. The average achieved fungal biomass with this *D* of 0.04 h^−1^ was 12.3 ± 2.2 g/L with an average biomass yield of 0.31 g/g of glucose and an average volumetric productivity of 0.48 g/L/h. In these fermentations, the residence time was 25 h and at a *D* of 0.04 h^−1^, the sustained residence time was achieved for over 180 h (Fig. 7).

### *D* 0.07 h^−1^ (with a 68-h batch phase)

**Fig. 8:**
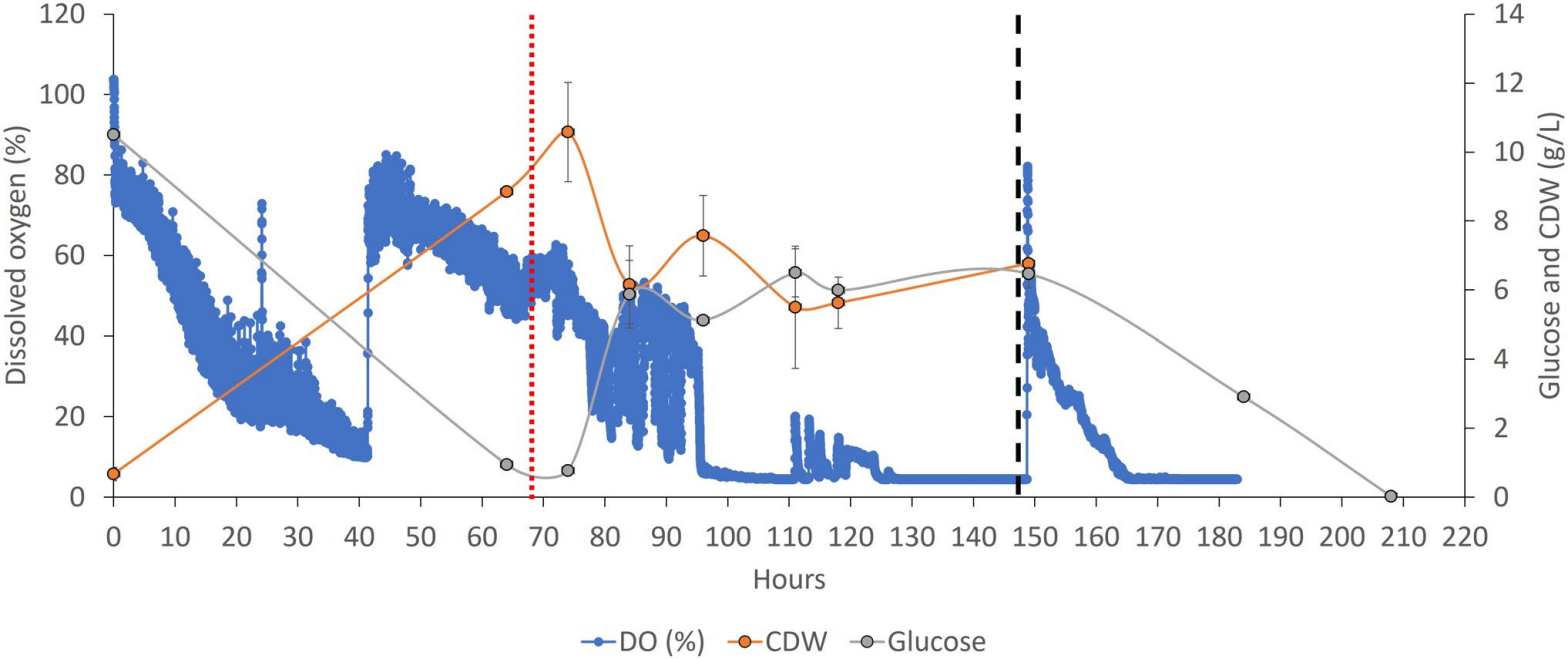
Average plot of triplicate semi-continuous fermentations targeting a dilution rate (*D*) of 0.07 h^−1^ for the growth of *T. versicolor* after a batch phase of 68 h. A vertical red dotted line identifies the start of the feeding regime. A vertical black dashed line identifies the onset of macroaggregate pellet formation

In order to improve the volumetric productivity further and decrease the residence time of the medium, we planned a set of triplicate semi-continuous fermentations targeting a dilution rate of 0.07 h^−1^. Conscious of the risk of the fungal biomass not able to cope with the dilution rate, we decided to extend the batch phase by a further 17–23 h, i.e. to 68 h. After a batch phase of minimum of 65 h and with a similar approach that tailored the starting of the semi-continuous fermentation after a more pronounced and sustained rise of the dissolved oxygen trend, the *D* of 0.07 h^−1^ induced a biomass growth that reached 7.5 g/L in the best scenario. A considerable drop in the recorded biomass value and a consequential rise in the glucose concentration were seen in both of the two more successful fermentations that attempted this dilution rate (Fig. 8). The CDW concentration of 10.6 g/L achieved at 74 h, following 68 h of the batch phase, decreased by 42% within the first 19 h of the semi-continuous phase (6.15 g/L) (Fig. 8). After this time, the fungal biomass recovered to some degree, reaching an average 7.5 g/L CDW. The semi-continuous fermentation at *D* = 0.07 h^−1^ lasted a total maximum of 150 h (Fig. 8). The average biomass yield, the average volumetric productivity and highest CDW, with this dilution rate, were 0.31 g of CDW/g of glucose, 0.52 g/L/h and 7.5 g/L respectively.

### *D* 0.1 h^−1^ (with a 71-h batch phase)

A dilution rate of 0.1 h^−1^ represents a situation where 10% of the medium is replaced every hour; this by definition means that the residence time is 10 h. A fungal strain of *T. versicolor* showed to have a generation time of 17 h. This is 7 h longer than the residence time; therefore, if the biomass is not actively growing in every pellet in its entirety and surface area, the risk of washout is considerably high. In spite of these challenging conditions, we planned a semi-continuous fermentation that tried to target this dilution rate, after a batch phase of 71 h. This was again intended, so the glucose consumption of the batch phase could be maximised. The biomass yield, volumetric productivity and maximum CDW achieved with this dilution rate were 0.46 g/g, 0.87 g/L/h and 8.7 g/L respectively (Table 2; Supplemental Fig. S2), which is only 1.8 times the residence time of 10 h; therefore, it cannot be said that the *T. versicolor* biomass maintained steady-state conditions for a sufficient time. Unfortunately, not enough biomass could be sampled for the analysis of the glucans at this *D* due to shorter time span and the higher washout. At dilution rates higher than 0.1 h^−1^, the fungal culture was unable to maintain a steady state in a semi-continuous culture (data not shown). A biomass of 1 g/L was achieved but could not be maintained, and while the glucose concentration during the early phase of the semi-continuous phase was consumed, the fungal biomass was washed out.

**Table 2:** Yields and performance parameters of the semi-continuous fermentations for the cultivation of *T. versicolor* as the fungal strain of choice

*D* (h^−1^)	Biomass yield (g/g)	CDW (g/L)	Volumetric productivity (g/L/h)	Sustained residence time (fold)
0.02	0.38	13.4	0.26	2.4
0.04	0.31	12.3	0.48	7.2
0.07	0.31	7.5	0.52	5.6
0.1	0.46	8.7	0.87	1.8

### Glucan accumulation

Glucan accumulation by *T. versicolor*, grown with a *D* of 0.02, 0.04 h^−1^, 0.07 h^−1^ and 0.1 h^−1^, showed striking differences (Supplemental Fig. S3). A *D* of 0.02 h^−1^ causes a decrease in total, α and β-glucans when the fungal biomass is at its maximum CDW (90 h) (Supplemental Fig. S3). At a *D* of 0.04 h^−1^ at T140 (maximum CDW), the total and *β-*glucan amounts are 1.6- and 5.2-fold higher, respectively, than at *D* of 0.02 h^−1^; possibly because at this optimal feeding regime, the growth rate is maximum, and the carbon source is taken up optimally. Both *D* of 0.07 and 0.1 h^−1^ show an increasing trend for total and *β-*glucans, especially in the early phase of the semi-continuous fermentation (between 71 and 96 h); in fact, the total and *β-*glucan content are 4.8- and 14.9-fold higher for a *D* of 0.07 h^−1^ and 3.4- and 8.7-fold higher for a *D* of 0.1 h^−1^ when compared to the same (T90) time point of a *D* of 0.02 h^−1^.

## Discussion

The growth of edible filamentous fungi in bioreactors as a source of bioactives and protein could offer new possibilities for human and animal nutrition (Colosimo et al. 2021). Edible fungi are well known to benefit human and animal health (Bozbulut and Sanlier 2019). Producing fungi in liquid media in bioreactors as opposed to using solid substrates could improve volumetric productivity (Patel et al. 2023). Continuous culture has been investigated as an effective method to have higher volumetric productivity of microbial biomass (Wang et al. 2013; Liu et al. 2019); to the best of our knowledge, not many of these studies have been undertaken for edible fungi (Jin et al. 2001; Thunuguntla et al. 2018). We investigated five well-known fungal strains. Clear metabolic differences were seen among the five different tested fungal strains. *T. versicolor* outperformed the growth rates of the other four fungal strains both in YMSG and CSL-media. *T. versicolor* behaves consistently both in rich and diminished media, showing a fast growth rate in the early exponential phase with a minimal adaptation phase (short lag). Thiruchelvam and Ramsay (2007) also saw a similar metabolic behaviour regarding the maximum growth of this strain. *H. erinaceus* also behaved similarly in both media but with a slightly lower growth rate than *T. versicolor*, similarly to what reported by Krzyczkowski et al. (2010) where *H. erinaceus* had a specific growth rate of 0.0075 h^−1^. We saw specific growth rates of 0.013 h^−1^ and 0.006 h^−1^ on rich media and on CSL media respectively. *A. blazei* and *L. edodes* showed a longer lag phase in rich media than in minimal media (CSL-based), and both have a remarkable growth difference correlated with the type of media, even if *L. edodes* showed a similar growth rate to the one reported by Garcia-Cruz et al. (2020). *A. bisporus* performed poorly in both media. A planned reduction of the components of the minimal medium (CSL-based) was intended as the logical progression of the study; this was to decrease media costs, with future scale-up in mind.

The removal of the primary carbon source (glucose) has the most severe impact as expected, but *H. erinaceus* and *L. edodes* were the most resilient strains to cope with this variation; this resilience was also highlighted by the studies of Imtiaj et al. (2008) and Garcia-Cruz et al. (2020), where an extreme pH tolerance was shown for *H. erinaceus* and *L. edodes* respectively. The concomitant or individual halving of glucose and yeast extract impacted severely on *T. versicolor* and *A. blazei*, possibly because these two strains are more affected by the reduction of the primary carbon source to fuel their growth (Berovic and Podgornik 2016). *L. edodes* is instead more affected by the reduction of CSL, possibly because critical components of this mixed substrate (possibly oligopeptides or single amino acids) cannot be biosynthesised by the metabolic machinery of this strain; in fact, Yu et al. (2023) highlighted a specific pattern of amino acid biosynthetic capability of *L. edodes*. As mentioned above, the most resilient strain in surviving the lack of primary carbon source (halved glucose) and secondary carbon source (CSL) is *H. erinaceus*; this strain shows a 25% reduction in CDW when grown under those conditions. Conversely, *H. erinaceus* is most severely affected in its ability to synthesise glucans (especially β-glucans). *L. edodes* increases its biosynthesis of b-glucans when glucose and yeast extract are halved or if CSL is completely removed. However, *T. versicolor* showed its maximum β-glucan production when yeast extract (YE) is completely removed or halved; it is possible that the removal of YE is stressful for the cells and that such a stressful condition promotes the enrichment of fungal biomass with this structural polysaccharide (in *L. edodes*, *A. blazei* and *T. versicolor*). A set of airlift-based batch experiments confirm that *T. versicolor* has both the highest substrate consumption, the highest *μ*_MAX_ and the highest β-glucans/total glucan ratio. Therefore, because of the overall performance of this strain, it was brought forward for semi-continuous fermentation studies. *T. versicolor* is able to maintain growth at a dilution rate of 0.04 h^−1^ over a 184-h period in semi-continuous mode before biomass clumping interferes with the growth of the organism. At a *D* of 0.02 h^−1^, *T. versicolor* achieves a higher biomass compared to cells grown at a *D* of 0.04 h^−1^, but mycelial clumping occurs earlier and terminates the fermentation at *T* 168 h (Fig. 6). Higher dilution rates (0.07 h^−1^) result in a decrease in total biomass (7.5 g/L vs 13.4 g/L) but allow a longer sustained residence time (80 h; Fig. 8; Table 2). The highest *D* (0.1 h^−1^) not only causes the highest volumetric productivity but also shows the shortest sustained residence time (18 h); a 10% replacement of the media every hour (i.e. a 10-h residence time) is too challenging for a strain that has a generation time of 17 h in batch cultivation.

Glucan production is somehow linked to the dilution rate. At a dilution rate of 0.02 h^−1^, the actively dividing hyphal apical cells do not accumulate high levels of glucans and are only able to accumulate substantial amounts of total glucans at the end of the batch phase or at the end of the semi-continuous fermentation where cells experience a reduction of glucose concentration. At a *D* of 0.04 h^−1^, significant accumulation also occurs at the end of the semi-continuous phase. While at a *D* of 0.07 h^−1^, an overfeeding of glucose (between 92.5 and 149 h) coincides with an increase of both total and β-glucans (Supplemental Fig. S3) but a shorter fermentation duration. In conclusion, while all five strains are able to grow in submerged fermentation and some strains accumulated higher levels of glucan than *T. versicolor*, the latter demonstrated the highest growth and thus the best overall biomass and glucan productivity in shaken flasks. Submerged cultivation of a *T. versicolor* fungal strain by a semi-continuous strategy using an airlift bioreactor allowed for fungal biomass production of up to 13.4 g/L. Tailoring the *D* to 0.04 h^−1^ achieved the overall best biomass/bioactives titre (12.3 g/L and 2.21 g/L), the highest volumetric productivities (0.48 g/L/h of biomass and 0.016 g/L/h of total glucans) and the maximum sustained residence time (180 h). The lowest *D* (0.02 h^−1^) allowed the highest CDW (13.4 g/L), but reduced volumetric productivities (0.26 g/L/h biomass and 0.01 g/L/h total glucans) to the one achieved with a *D* to 0.04 h^−1^, and the residence time cannot be sustained for as long as with a *D* of 0.04 h^−1^. Conversely, a higher *D* (0.07 h^−1^) achieved a lower CDW (7.5 g/L), comparable volumetric productivities for both biomass and total glucans (0.52 g/L/h and 0.02 g/L/h respectively) when compared with the values obtained with a *D* of 0.04 h^−1^, but the residence time cannot be sustained for as long as with a *D* of 0.04 h^−1^.

## Supplementary Information

Below is the link to the electronic supplementary material.

Supplementary file1 (PDF 289 KB)

## Data Availability

The authors confirm that the datasets supporting the findings and conclusions of this study are available within the article. Additional data can be provided upon request.
