# Supplementary material for: Airlift bioreactor–based strategies for prolonged semi-continuous cultivation of edible Agaricomycetes
Source: Appl Microbiol Biotechnol. 2024 Jun 18;108:377. doi: 10.1007/s00253-024-13220-4 (PMC11189342; doi:10.1007/s00253-024-13220-4)
Supplement: Supplementary file 1 [file 253_2024_13220_MOESM1_ESM.pdf]

Applied Microbiology and Biotechnology – Supplemental material.

Air-lift bioreactor-based strategies for prolonged semi-continuous cultivation of edible *Agaricomycetes*

Federico Cerrone[1,2], Conor Ó Lochlainn[1,2], Tony Callaghan[3], Peter McDonald[3], Kevin O'Connor[1,2,4*]

[1]BiOrbic Bioeconomy Research Centre, O'Brien Centre for Science (Science East) University College Dublin, Belfield Campus Dublin, Ireland

[2]School of Biomolecular and Biomedical Sciences University College Dublin, Belfield Campus Dublin, Ireland

[3]Commercial Mushroom Producers, Units7/8 Newgrove Industrial Estate, Monaghan, Ireland

[4] Bioplastech Ltd NovaUCD, Belfield Innovation Park, University College Dublin, Ireland

*Kevin.oconnor@ucd.ie, telephone and fax number +353(1)7162198

Supplementary Figure S1

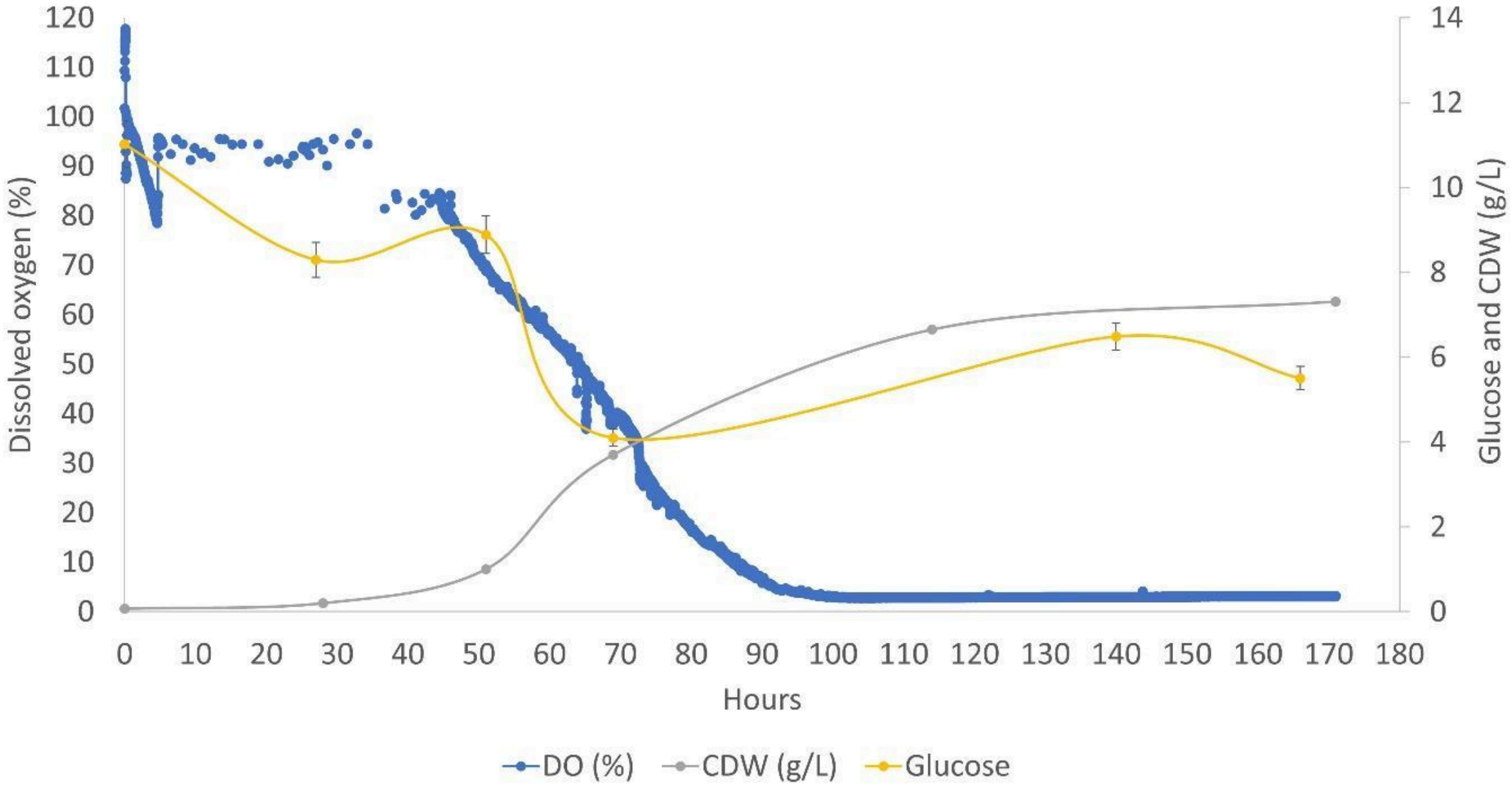

Supplementary Figure S1. Semicontinuous fermentation targeting a dilution rate (D) of 0.04 h^{-1} for the growth of *T. versicolor* after a batch phase of 31 h.

Supplementary Figure S2

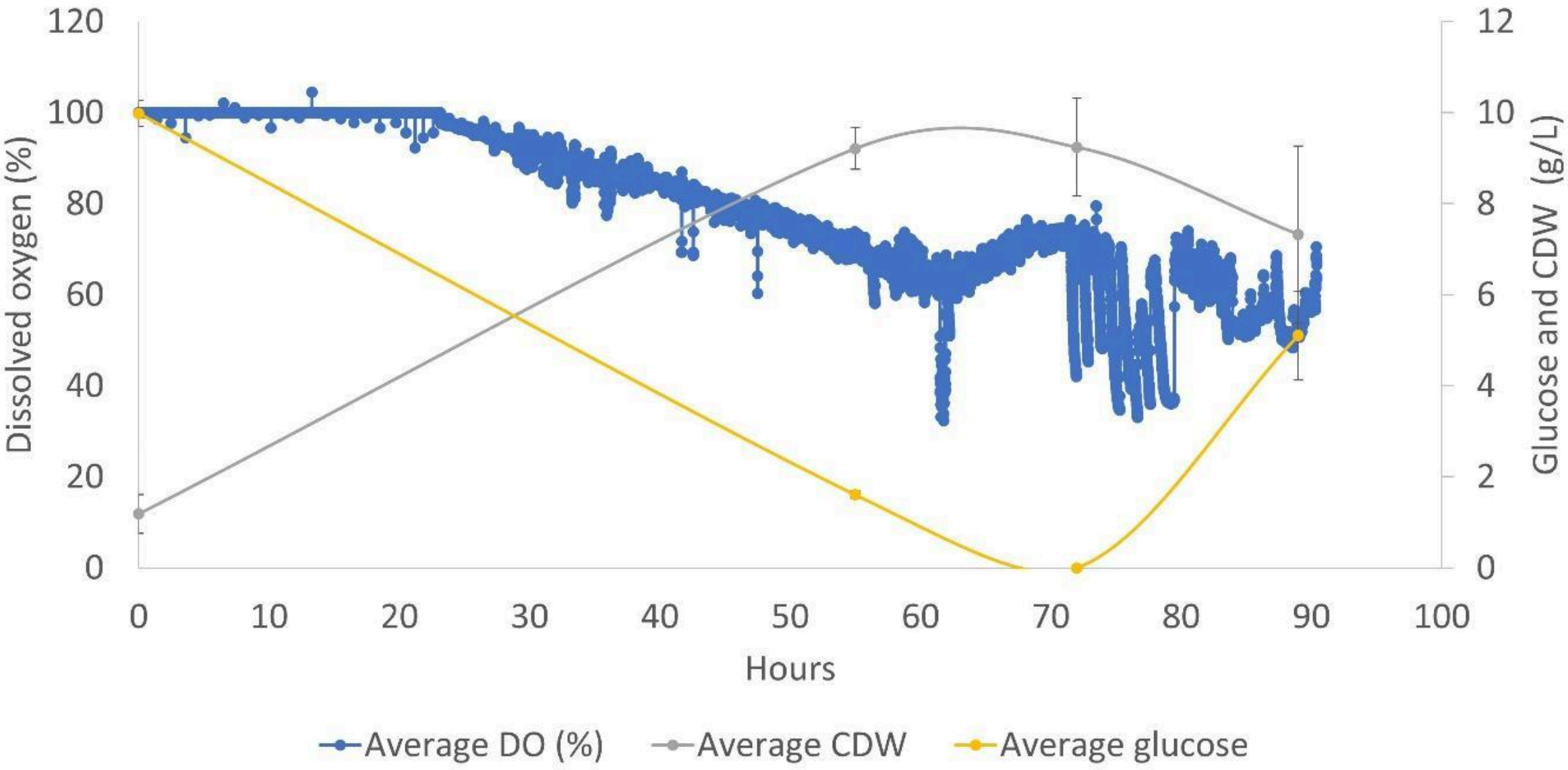

Supplementary Figure S2. Semicontinuous fermentation targeting a dilution rate (D) of 0.1 h^{-1} for the growth of *T. versicolor* after a batch phase of 71 h.

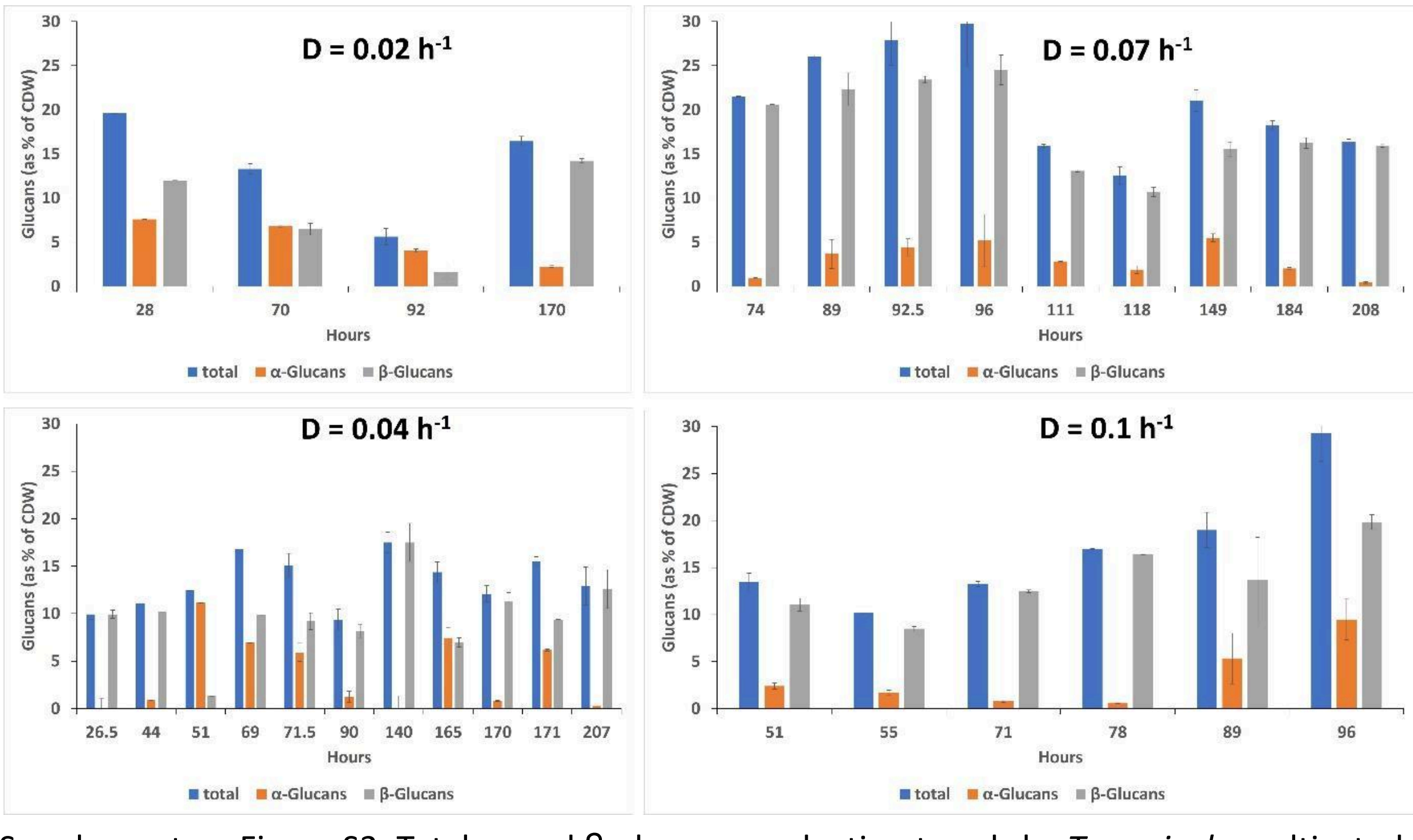

Supplementary Figure S3. Total, α and β-glucans production trends by *T. versicolor* cultivated in semi-continuous fermentation with different dilution rates, D (namely, D of 0.02, 0.04, 0.07 and 0.1 h^{-1})
